# Supplementary material for: Cooperativity in Plant Plasma Membrane Intrinsic Proteins (PIPs): Mechanism of Increased Water Transport in Maize PIP1 Channels in Hetero-tetramers
Source: Sci Rep. 2018 Aug 13;8:12055. doi: 10.1038/s41598-018-30257-4 (PMC6089885; doi:10.1038/s41598-018-30257-4)
Supplement: Supplementary file 1 — Supplementary Information [file 41598_2018_30257_MOESM1_ESM.doc]

**SUPPORTING INFORMATION**

**Cooperativity in Plant Plasma Membrane Intrinsic Proteins (PIPs): Mechanism of Increased Water Transport in Maize PIP1 Channels in Hetero-tetramers**

**Manu Vajpai**^1^**^#^, Mishtu Mukherjee**^1^**^#^ and R. Sankararamakrishnan**^1^*****

^1^*Department of Biological Sciences and Bioengineering, Indian Institute of Technology Kanpur, Kanpur – 208016, India*

**Figure S1**

**
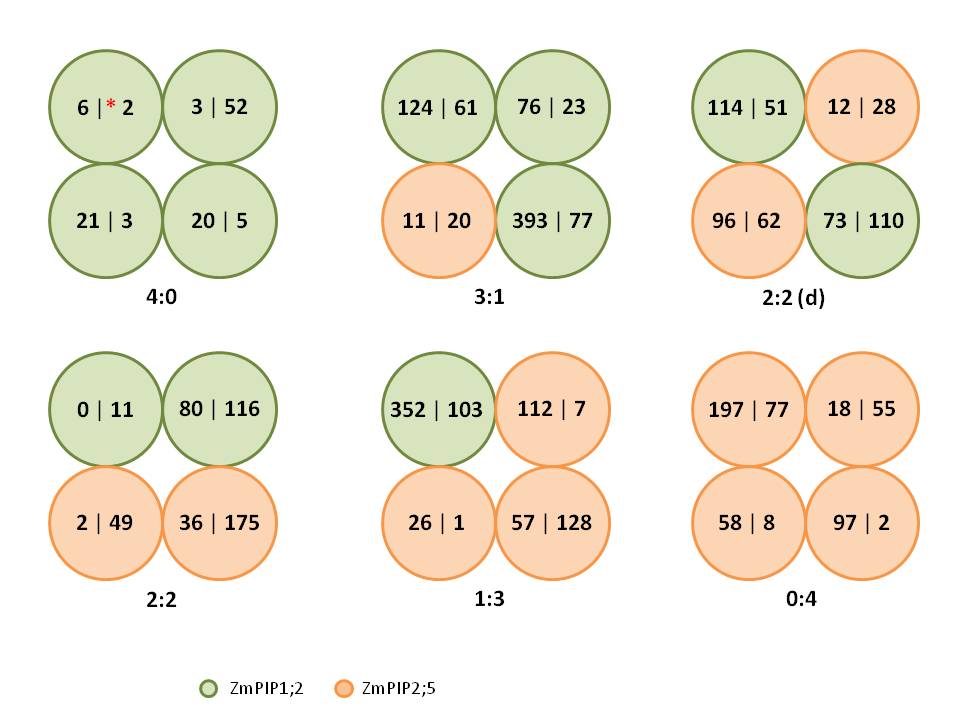
**

**Figure S1:** Number of observed water permeation events for each of the six simulated systems of ZmPIP1;2 and ZmPIP2;5 homo- and heterotetramers. The two values for each monomer separated by “|” represent the values obtained from two independent simulations.

**Figure S2**


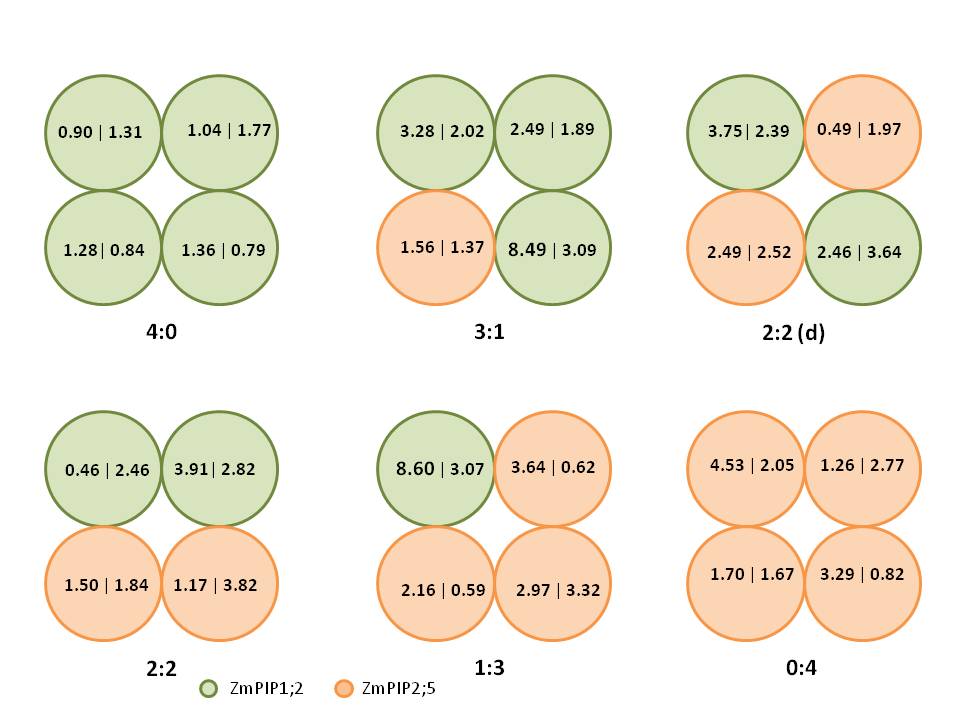


**Figure S2:** Osmotic permeability (pf) values for each of the six simulated systems of ZmPIP1;2 and ZmPIP2;5 homo- and hetero-tetramers. The two values for each monomer separated by “|” represent the values obtained from two independent simulations.

**Figure S3**


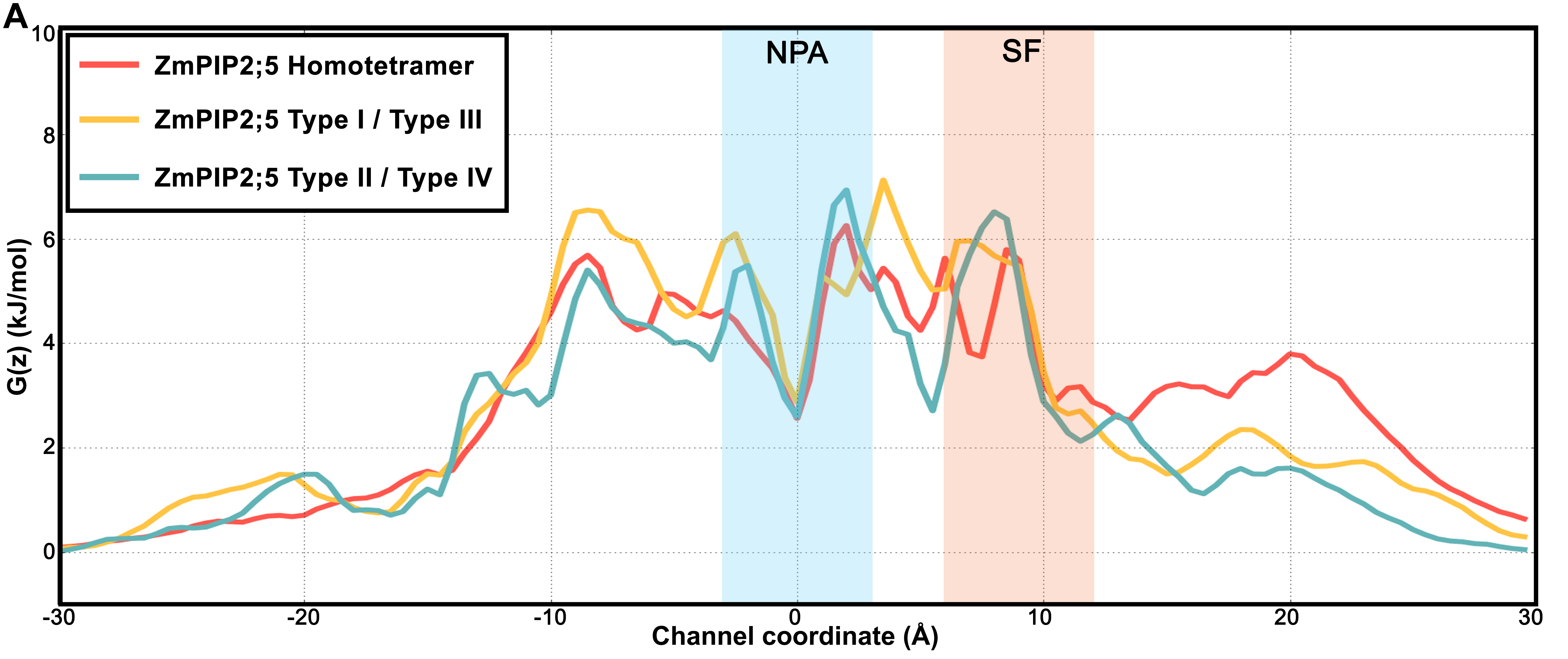


**Figure S3**: Average potential of mean force (PMF) profiles calculated for ZmPIP2;5 monomers from homotetrameric system (4 monomers from 0:4 system), ZmPIP2;5 monomers belonging to Type I/Type III (3 monomers from 1:3 and 2:2 systems) and Type II/Type IV (5 monomers from 1:3, 2:2, 2:2(d) and 3:1 systems) groups. Definition of Type-I to Type-IV monomers for ZmPIP2;5 system is similar to that used for ZmPIP1;2 monomers as given in the main manuscript. MD trajectories from both independent simulations were merged to calculate the average PMF profiles. The regions corresponding to the aromatic/arginine selectivity filter (SF) and the conserved NPA motif (NPA) are indicated.

**Figure S4**


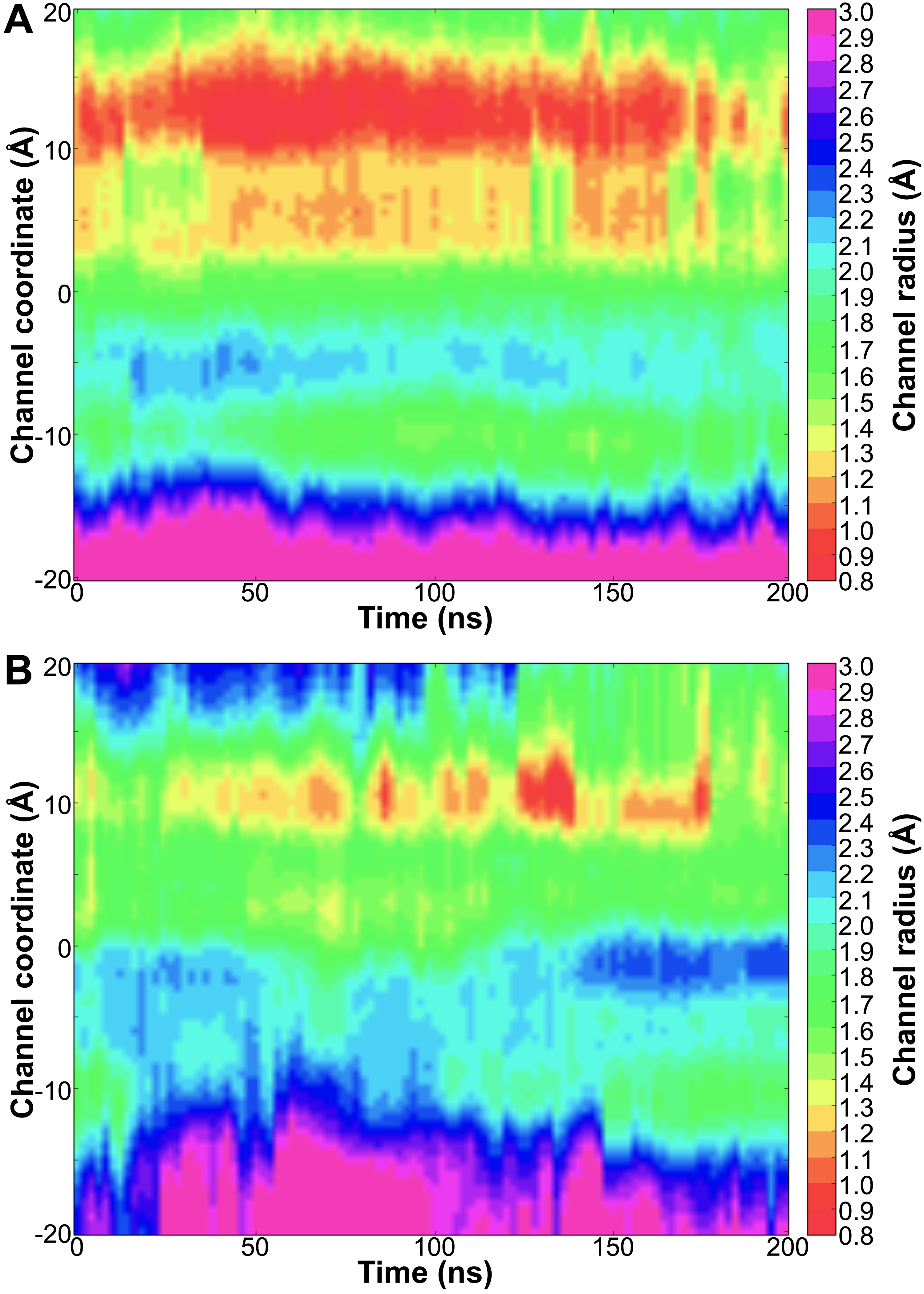


**Figure S4**: Temporal channel radius profiles of (A) ZmPIP1;2 with Type III-like interface from 2:2 (d) mutant system (2 monomers) and (B) ZmPIP1;2 with Type IV interface from 2:2 (d) mutant system (2 monomers). The temporal channel radius profiles for monomers of the same type were calculated as described in the Methods section.

**Figure S5**


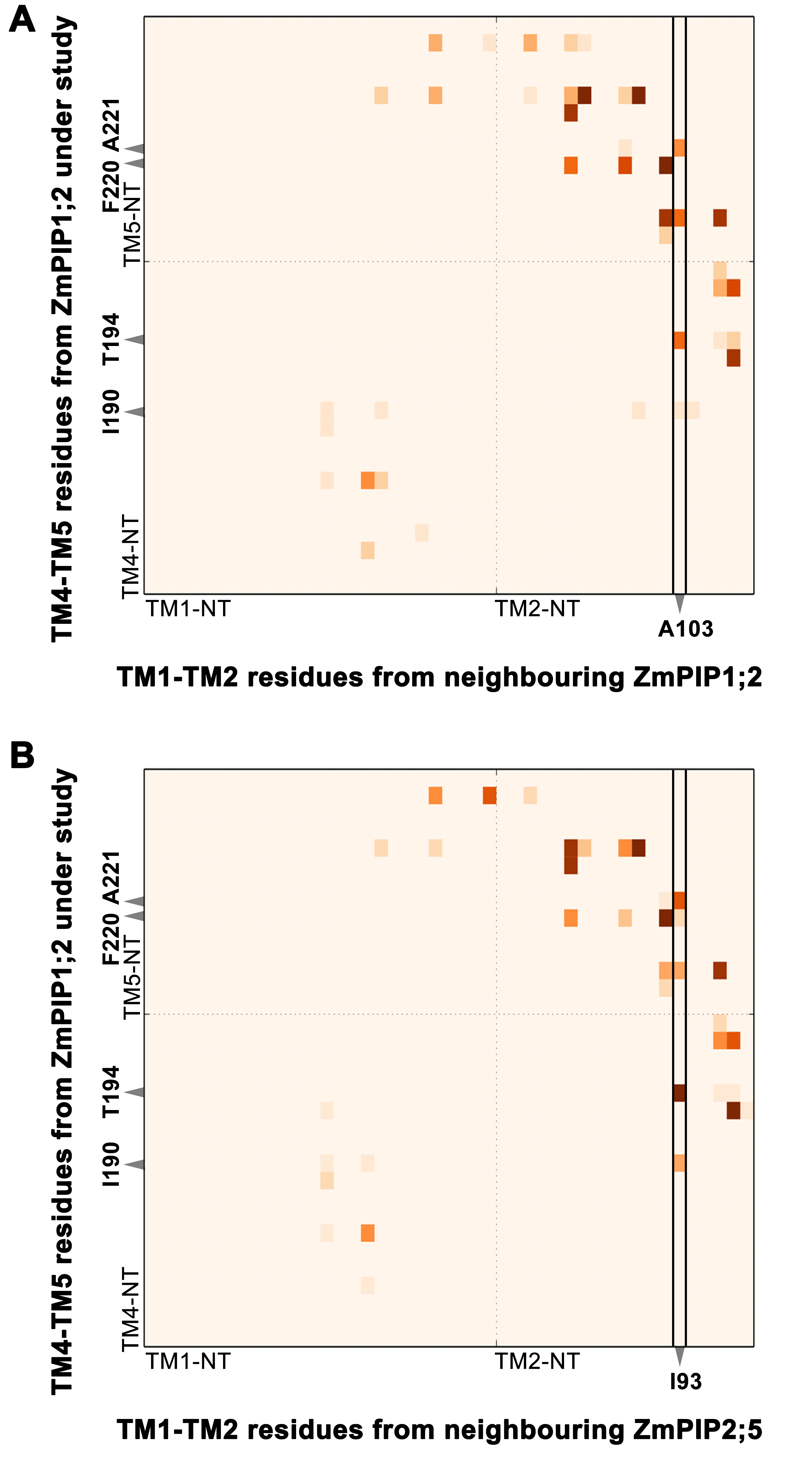


**Figure S5:** (A) Contact map of ZmPIP1;2 monomers in the homo-tetramer system. Contacts between residues at the interface of TM1-TM2 and TM4-TM5 regions are plotted. Contacts were calculated for the interfaces of all four monomers and normalized. (B) Contact map calculated for all ZmPIP1;2 monomers that belong to Type II/Type IV category in all the hetero-tetramer systems. This plot represents the normalized contacts between residues of TM4-TM5 region of ZmPIP1;2 and TM1-TM2 segments of ZmPIP2;5. Important residues involved in contacts are marked in both (A) and (B).

**Figure S6**


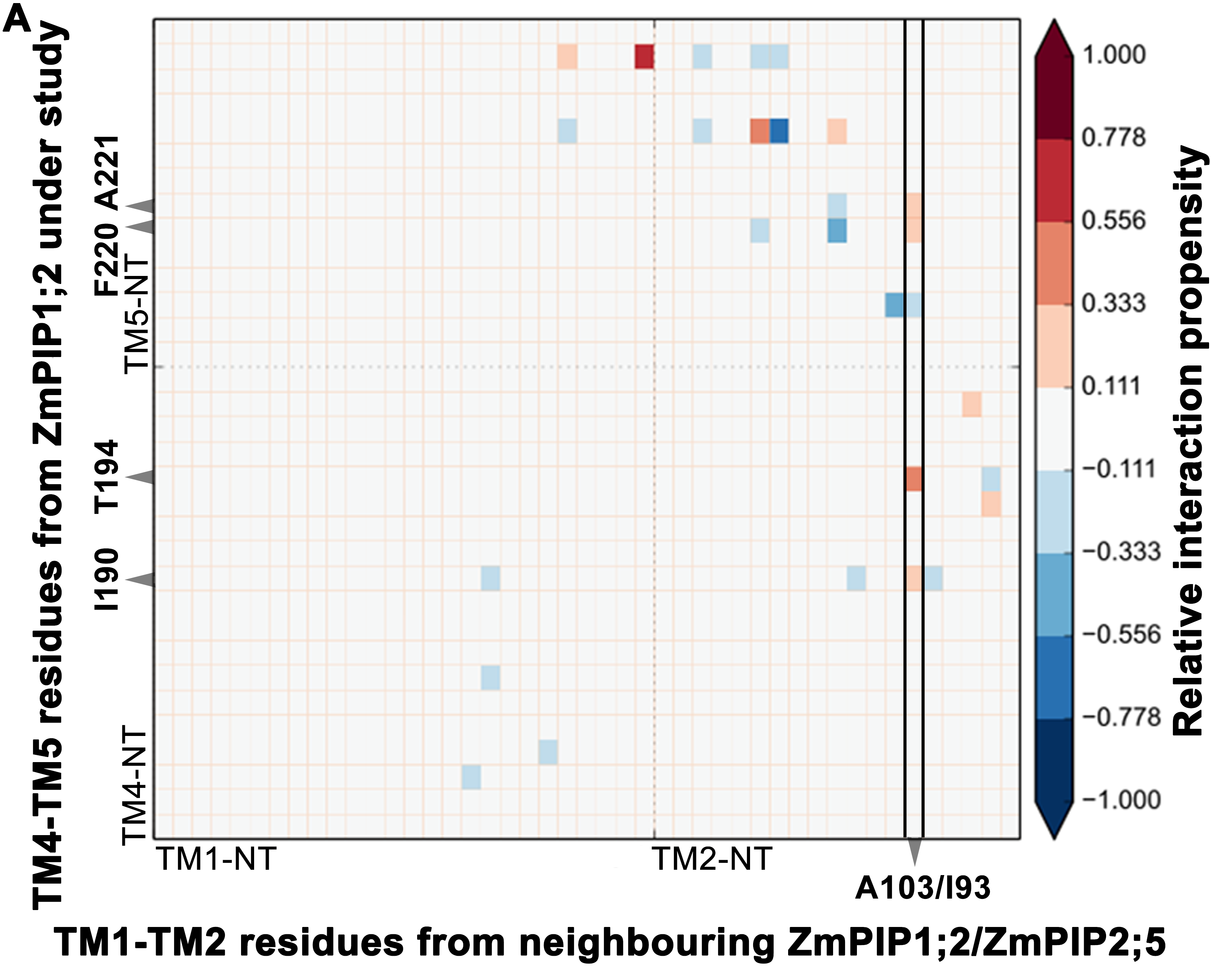


**Figure S6:** Difference contact map calculated for two types of ZmPIP1;2 monomers. In this analysis, one face of ZmPIP1;2 is TM4-TM region and that is same for all ZmPIP1;2 monomers. Its interacting region will be TM1-TM2 segments of either ZmPIP1;2 or ZmPIP2;5. Contact maps calculated for ZmPIP1;2 monomers from homo-tetramers (***TM4-TM5(I)****/TM1-TM2(I)* interface) and ZmPIP1;2 that belong to Type II/Type IV category (***TM4-TM5(I)****/TM1-TM2(II)* interface) were used to calculate the difference contact map. The scale indicates the “Relative interaction propensity” with blue being low and red being high propensity to make contacts. Contacts involving I93 of ZmPIP2;5 residue clearly indicates high interaction propensity with multiple residues of ZmPIP1;2.

**Figure S7**


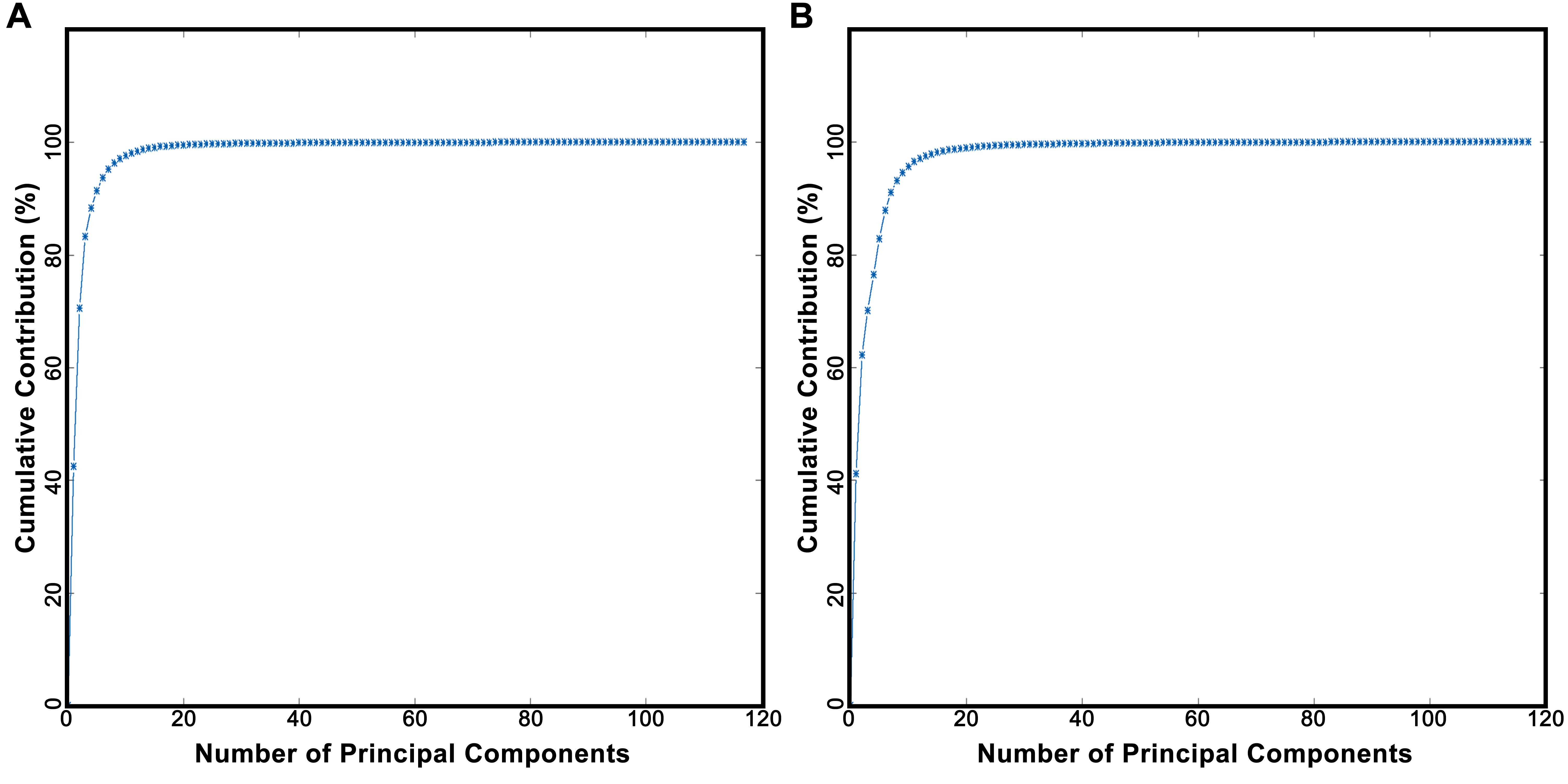


**Figure S7:** Cumulative contribution of eigenvectors to the overall motion of the channels under consideration. Cumulative contribution of (A) ZmPIP1;2 monomers from homotetramers and (B) ZmPIP2;5 monomers of Type II/Type IV category from hetero-tetramers. About 80% of the total mean square fluctuations can be described by the first 10 eigenvectors. The first eigenvector alone represents more than 40% of total fluctuations.
